# Schwann cell-derived exosomes ameliorate peripheral neuropathy induced by ablation of dicer in Schwann cells

**DOI:** 10.3389/fncel.2024.1462228

**Published:** 2024-09-02

**Authors:** Lei Wang, XueRong Lu, Alexandra Szalad, Xian Shuang Liu, Yi Zhang, Xinli Wang, William Anthony Golembieski, Brianna Powell, Mikkala Mccann, Mei Lu, Michael Chopp, Zheng Gang Zhang

**Affiliations:** ^1^Department of Neurology, Henry Ford Hospital, Detroit, MI, United States; ^2^Department of Biostatistics and Research Epidemiology, Henry Ford Hospital, Detroit, MI, United States; ^3^Department of Physics, Oakland University, Rochester, MI, United States

**Keywords:** dicer, Schwann cells, exosomes, microRNAs, peripheral neuropathy, mice

## Abstract

**Background:**

MicroRNAs (miRNAs) in Schwann cells (SCs) mediate peripheral nerve function. Ablating Dicer, a key gene in miRNA biogenesis, in SCs causes peripheral neuropathy. Exosomes from healthy SCs (SC-Exo) ameliorate diabetic peripheral neuropathy in part via miRNAs. Thus, using transgenic mice with conditional and inducible ablation of Dicer in proteolipid protein (PLP) expressing SCs (PLP-cKO), we examined whether SC-Exo could reduce peripheral neuropathy in PLP-cKO mice.

**Methods:**

PLP-cKO mice at the age of 16 weeks (8 week post-Tamoxifen) were randomly treated with SC-Exo or saline weekly for 8 weeks. Age-and sex-matched wild-type (WT) littermates were used as controls. Peripheral neurological functions, sciatic nerve integrity, and myelination were analyzed. Quantitative RT-PCR and Western blot analyses were performed to examine miRNA and protein expression in sciatic nerve tissues, respectively.

**Results:**

Compared to the WT mice, PLP-cKO mice exhibited a significant decrease in motor and sensory conduction velocities, thermal sensitivity, and motor coordination. PLP-cKO mice exhibited substantial demyelination and axonal damage of the sciatic nerve. Treatment of PLP-cKO mice with SC-Exo significantly ameliorated the peripheral neuropathy and sciatic nerve damage. PLP-cKO mice showed a substantial reduction in a set of Dicer-related miRNAs known to regulate myelination, axonal integrity, and inflammation such as miR-138, −146a and − 338 in the sciatic nerve. In addition, PLP-cKO mice exhibited significant reduction of myelin forming proteins, early growth response 2 (EGR2) and sex determining region Y-box10 (Sox10), but significantly increased myelination inhibitors, Notch1, c-Jun, and Sox2 and the axonal growth inhibitor phosphatase and tens in homolog (PTEN). However, SC-Exo treatment reversed the PLP-cKO altered miRNAs and proteins.

**Conclusion:**

This study demonstrates that exogenous SC-Exo ameliorate peripheral neuropathy induced by Dicer ablation in PLP expressing SCs. The therapeutic benefit may be mediated by the SC-Exo altered miRNAs and their targeted genes.

## Introduction

Schwann cells (SCs) are the principal glial cells in the peripheral nervous system (PNS), playing pivotal roles in the development and maintenance of peripheral nerve function (Jessen et al., 2015; Castelnovo et al., 2017). Proteolipid protein (PLP) is a major myelin protein that is specifically expressed in oligodendrocytes in the central nervous system (CNS) and in SCs in the peripheral nervous system (PNS) (Kamholz et al., 1992; Garbern et al., 1997). Investigating PLP provides valuable insights into myelin biology and interactions between myelin proteins in both the CNS and PNS, and may facilitate identification of novel therapeutic strategies for demyelinating diseases and peripheral neuropathy.

MicroRNAs (miRNAs) in SCs are important regulators for myelination and axonal integrity in the PNS (Pereira et al., 2010; He et al., 2012; Borger et al., 2022). Dicer is a key gene for miRNA biogenesis, responsible for processing pre-miRNAs into mature miRNAs (Pereira et al., 2010; Koscianska et al., 2011). The ablation of Dicer selectively in SCs, including SCs expressing PLP, results in a significant decrease in Dicer-related miRNAs, and impairments of development and maintenance of myelination and axonal integrity, leading to peripheral neuropathy(Pereira et al., 2010; Viader et al., 2011; Gokbuget et al., 2018; Li et al., 2018). For example, Gokbuget et al. (2018) demonstrated that the ablation of Dicer in P0 lineage SCs disrupts the maintenance of myelination. Li et al. (2018) showed that adult mice with the inducible knockout of Dicer in PLP expressing SCs exhibit severe sciatic nerve degeneration, and peripheral motor dysfunction and paralysis.

Exosomes, small extracellular vesicles, play a pivotal role in facilitating cell–cell communication to mediate recipient cell biological function by transporting their cargo, including miRNAs and proteins, to recipient cells (Bang and Thum, 2012; Lai and Breakefield, 2012). Emerging studies on animal models of peripheral nerve injury, spinal cord injury, traumatic brain injury, and chemo-induced neuropathy show that SC-Exo have therapeutic effects on nerve regeneration, angiogenesis, and alleviation of neuropathy (Lopez-Verrilli et al., 2013; Huang et al., 2022; Liu et al., 2022; Ren et al., 2023; Sun et al., 2023; You et al., 2023; Zhu et al., 2024). Recently, allogeneic SC-Exo have been investigated in treating a patient with amyotrophic lateral sclerosis (Goldschmidt-Clermont et al., 2025). We previously demonstrated that exosomes derived from high-glucose-stimulated SCs promote the development of diabetic peripheral neuropathy (DPN) (Jia et al., 2018), whereas, treatment of DPN mice with exosomes derived from healthy SCs (SC-Exo) ameliorates DPN by increasing intraepidermal nerve fibers and reducing axonal and myelin damage of the sciatic nerve (Wang et al., 2020). Intravenously administered SC-Exo alter miRNAs within the sciatic nerve of DPN mice, suggesting that SC-Exo cargo miRNAs contribute to the therapeutic effect of SC-Exo on DPN (Wang et al., 2020). However, whether SC-Exo provide the therapeutic benefit for peripheral neuropathy induced by deletion of Dicer specifically in SCs has not been investigated. Therefore, using transgenic mice with a conditional and inducible ablation of Dicer in PLP expressing SCs (PLP-cKO), we tested the hypothesis that treatment of young adult PLP-cKO mice with SC-Exo reduces peripheral neuropathy.

## Materials and methods

### Animals

All animal experimental procedures were approved by the Institutional Animal Care and Use Committee of Henry Ford Hospital (IACUC #1245) and were performed in compliance with NIH Guidelines for the Care and Use of Laboratory Animals.

### Generation of PLP-reporter mice and PLP-cKO mice

To investigate whether SC-Exo can rescue Schwann cell ablation of Dicer induced peripheral neuropathy in adult mice, we elected the PLP-Cre^ERT2^ mouse line, which permits us to generate conditional and inducible ablation of Dicer in PLP expressing SCs in adult mice after breeding with Dicer*^fl/fl^* mice. To verify PLP expressing SCs, a PLP-reporter line was generated by crossing transgenic mice expressing Cre recombinase under the control of the PLP promoter (PLP-Cre^ERT2^, stock#: 005975, JAX) with Rosa-TdTomato mice (stock# 007914, JAX). To generate PLP-cKO mice (PLP-Cre^ERT2^:Dicer^fl/fl^ mice), PLP-Cre^ERT2^ mice were crossed to mice with a floxed allele of Dicer (Dicer*^fl/fl^* stock#: 006001, JAX). Homozygous female and male PLP-Cre^ERT2^:Dicer^fl/fl^ mice confirmed by RT-PCR genotyping were used in the present study. PLP-reporter mice and PLP-Cre^ERT2^:Dicer^fl/fl^ mice at age of 8 weeks were intraperitoneally administered tamoxifen (TAM, 100 mg/kg/d, dissolved in 20 mg/mL sunflower seed oil) for 5 consecutive days, and these mice are referred to as PLP-reporter and PLP-cKO mice, respectively. Age and sex matched wild-type (WT) litters treated with TAM were used as controls. Female and male mice were used for all experiments.

### Isolation and characterization of SC-Exo

Mouse primary SCs were purchased from ScienCell (M1700-57), which were originally harvested from neonatal C57BL/6 mouse sciatic nerves. These cells have been well characterized morphologically and phenotypically, and exhibit positive expression of S100, GFAP and CD9. SCs at passage 3–5 were cultured in SC medium (1701, ScienCell). For the isolation of exosomes, when SCs reached a confluence of 60–80%, the culture medium was replaced with 5% exosome-depleted fetal bovine serum-contained medium (SBI System Bioscience) for an additional 48 h. Subsequently, medium was collected for SC-Exo isolation using a differential ultracentrifugation method, as previously described (Wang et al., 2020). Briefly, the supernatant was passed through a 0.22 μm filter to eliminate cellular debris and dead cells. A 10,000 g centrifugation for 30 min was conducted to further remove small debris. Ultracentrifugation was conducted at 100,000 g (Optima XE-100 Ultracentrifuge, SW 32 Ti Rotor) for 2 h. The pellet was resuspended with 100 μl of sterilized phosphate-buffered saline. Exosomes were characterized according to the guideline of Minimal information for studies of extracellular vesicles 2018 (MISEV2018) and our published studies (Thery et al., 2018; Wang et al., 2020). Briefly, Western blot analysis was performed for measuring exosomal marker proteins, Alix, CD9 and CD81, and for the negative exosomal marker protein Calnexin, an endoplasmic reticulum resident protein. Additionally, the NanoSight analysis system (Malvern Panalytical) was employed for quantification of exosome number and size.

### Experimental protocols

Female and male mice at the age of 16 weeks (8 week post TAM) were randomly assigned into following groups: (1) WT + saline (*n* = 12 with 6/sex). (2) PLP-cKO + saline (*n* = 12 with 6/sex), (3) PLP-cKO + SC-Exo (*n* = 12 with 6/sex). SC-Exo (2×10^10^ particles in 0.2 mL saline per injection) or equal volume of saline were intravenously administered weekly for 8 consecutive weeks.

Peripheral neurological function and electrophysiological measurements were performed every two and 4 weeks, respectively, for 8 weeks starting at 8 weeks post TAM. All mice were sacrificed after the 8 week SC-Exo treatment at mouse age of 24 weeks. The sciatic nerve tissues were collected for histopathological analysis and for assessments of miRNAs and proteins.

### Electrophysiological measurements of motor nerve conduction velocity (MCV) and sensory nerve conduction velocity (SCV)

MCV and SCV were assessed according to our published protocols (Wang et al., 2012; Wang et al., 2020). Briefly, mice were anesthetized with 1.5% isoflurane and electrodes were placed at the knee and sciatic notch. Trigger single square wave current impulses were delivered using a pulse stimulator. The simultaneous electromyography was recorded by two sterilized electrodes placed into the intrinsic muscle with a 4-channel amplifier (Natus UltraPro S100 EMG/NCS/EP Neurodiagnostic System). The temperature of mice was kept constant at 37°C ± 1.0°C during measurements using a water-based heater. MCV and SCV were calculated using the Natus Elite software provided by the manufacturer (Wang et al., 2020).

### Measurement of thermal sensitivity

Plantar test (Hargreaves Method) was conducted using a thermal stimulation meter (Model 336 TG, IITC Life Science), as previously described (Wang et al., 2012; Wang et al., 2020). Briefly, mice were acclimated on a transparent glass surface for a minimum of 20 min. To evaluate thermal sensitivity, a stimulator was placed beneath the hind paw’s plantar surface. The withdrawal latency in response to radiant heat at a 15% intensity, was recorded. Each animal was subjected to three readings, spaced 15 min apart. Subsequently, the average reading per mouse was calculated.

### Measurement of motor coordination and balance

Beam walking test was conducted as previously reported with some modifications (Lin et al., 2016). Briefly, mice were placed on a 90 cm x 1 cm x 1 cm wooden stick in a dark environment, with a light 50 cm away from the beam. The number of rear foot slips as each mouse crossed the 80 cm beam was quantified.

### Immunohistochemistry and image quantification

For immunohistochemistry, sciatic nerve tissues were fixed in 4% paraformaldehyde. The teased fibers and transverse sections (6 μm thick) were immunostained with chicken polyclonal anti-red fluorescent protein antibody (RFP, 1:200, Rockland antibodies and Assays), rabbit monoclonal antibody against S100 (1:400, Dako North America), rabbit polyclonal antibodies against myelin basic protein (MBP, 1:400, Dako North America, Inc.), chicken polyclonal antibodies against neurofilament heavy chain (NF-H,1:500 Thermo Scientific), respectively. Three fields of view per immunoreactive section were randomly acquired under a 40x objective (Carl Zeiss Axiostar Plus Microscope). The percentage of MBP and NF-H immunoreactive areas within the total imaged areas were calculated.

For analysis of intraepidermal nerve fibers (IENFs), footpad tissues were fixed in Zamboni’s fixative, and then cryosections (20 μm-thick) were stained with polyclonal antibody against protein gene product 9.5 (PGP9.5, 1:1,000; MILLIPORE). PGP9.5 immunoreactive fibers were imaged under a 40x objective (Carl Zeiss, Inc). The number of nerve fibers crossing the dermal-epidermal junction were measured and IENF density are presented as number of fibers per millimeter of length of epidermis. Representative IENFs were imaged with a laser scanning confocal microscope (Olympus Corporation) (Wang et al., 2015; Wang et al., 2020).

For analysis of morphometric changes of sciatic nerve, the nerve tissues were fixed in 2.5% glutaraldehyde and 0.5%sucrose in PBS, and then immersed in 2% osmium tetroxide for 2 h. The semi-thin transverse sections (2 μm thick) of sciatic nerve stained with toluidine blue were randomly imaged using a 100x oil immersion lens (Olympus Optical Co, Ltd). Myelinated fibers, axon diameter, and myelin sheath thickness were measured (Wang L. et al., 2011; Wang et al., 2020). Image analysis was performed using the MicroComputer Imaging Device imaging system (MCID, Imaging Research Inc).

### Transmission electron microscopy (TEM)

Mice were transcranial perfused with Ames’ medium containing heparin which was pre-oxygenated with a mixture of 95% O2, 5%CO2, and warmed to 37°C to remove blood. Subsequently, perfusion was carried out using a buffer containing 0.15 M sodium cacodylate (Electron Microscopy Sciences) (pH7.4) and 0.04% CaCl2 (MilliporeSigma), forming a cacodylate buffer. This buffer was supplemented with 2.5% glutaraldehyde (Electron Microscopy Sciences) and 2% paraformaldehyde, both warmed to 37°C. Tissues were dissected out and then post-fixed in the same fixative at 4°C overnight (Zhang et al., 2019). Then, tissues were dehydrated in graded alcohols and embedded in epoxy and cut into 1um sections. Sections were stained with uranyl acetate and lead citrate. Images were taken under the transmission electron microscope (JEM-1500 Flash, JEOL).

All analysis was conducted with the examiner blinded to the identity of the samples being studied.

### Quantitative real time RT-PCR (qRT-PCR)

Total RNAs from SC-Exo and sciatic nerve tissues were isolated using miReasy Mini Kit (Qiagen), followed by reverse transcription (RT). qRT-PCR was performed using a Taq-Man miRNA assay kit and amplified with Taqman PCR reagents, as previously described (Wang et al., 2012; Wang et al., 2020). Relative quantities of miRNAs were calculated by the 2-∆∆Ct method (Livak and Schmittgen, 2001) with U6 snRNA (Applied Biosystem) as the endogenous control. The primers used are given in Supplementary Table S1.

### Bioinformatics analysis

Signaling pathway analysis was conducted using Ingenuity Pathway Analysis (IPA) software (QIAGEN). Gene and miRNA names were imported into IPA and analyzed using the core analysis tool. Using the IPA knowledge base, networks of experimental proteins and miRNAs were constructed (Wang et al., 2023).

### Western blotting analysis

Western blot analysis was performed, as previously reported (Wang et al., 2012; Wang et al., 2020). Briefly, samples were homogenized using lysed buffer (RIPA, Sigma-Aldrich). Protein concentration in supernatant was determined with a bicinchoninic acid (BCA) assay kit (Pierce Biotechnology). From each sample, fifty micrograms of total protein were separated through 10% SDS-PAGE and transferred onto Polyvinylidene difluoride (PVDF) membranes. After blocking, the membranes were incubated with primary antibody overnight at 4°C. After incubation with secondary antibodies (1:1,000), an enhanced chemiluminescence development kit (Pierce Biotechnology) was used to detect the signals by FlurorChem E System (ProteinSimple). Individual primary antibodies used are provided in Supplementary Table S2.

### Primary culture of DRG neurons and evaluation of neurite outgrowth

DRG neurons were harvested from PLP-cKO mice and WT mice at the age of 24 weeks. Cultures were prepared according to published procedures (Wang et al., 2020). Briefly, DRG neurons were removed and dissociated by a Hanks balance salt solution (HBSS) containing 0.125% trypsin and 0.1% collagenase–A digestion for 30 min. Isolated DRG neurons were seeded on glass coverslips coated with laminin and poly-D-lysine in Neurobasal-A medium (Invitrogen, United States), 2% B-27 (GIBCO, United States). To evaluate the direct effect of SC-Exo on neurite outgrowth of DRG neurons, DRG neurons were cultured in DRG culture medium with or without SC-Exo (6×10^9^ particle/ml). After a 3-day culture, DRG neurons were immunostaining with antibody against neurofilament heavy subunit (NF-H, 1:500) for neurite outgrowth measurement. The images were captured at 10x magnification with a digital camera. Neurite outgrowth was measured in 20 neurons per coverslip. The total neurite lengths of each positive neuron were measured using MCID analysis system.

### Statistical analysis

All analyses were performed using SAS (9.4) and GraphPad 8 (version 8.2.1). Function data were assessed for normality, and data transformation was considered if data were not normally distributed. The repeated measure analysis of variance (ANCOVA) was used to study-group effect on each functional tests measured at each time point, including baseline change over time. The analysis first tested sex by group interaction, followed by testing group by time interactions, as well as the subgroup analyses. The study primarily focused on the SC-Exo effect on PLP-cKO mice. WT controls were included as the comparison group to assess functional deficits after PLP-KO. A significant SC-Exo by time interaction indicated that the effects of SC-Exo varied across time. The one-way analysis of variance (ANOVA), followed by Tukey’s multiple comparisons test were performed when comparing more than two group. Two-tailed Student’s t-test was used for two-group comparisons. The mean ± standard error (SE) and significant difference of two groups (*p*-value <0.05) were plotted for illustration.

## Results

### PLP-cKO mice develop peripheral neuropathy

**Figure 1:**
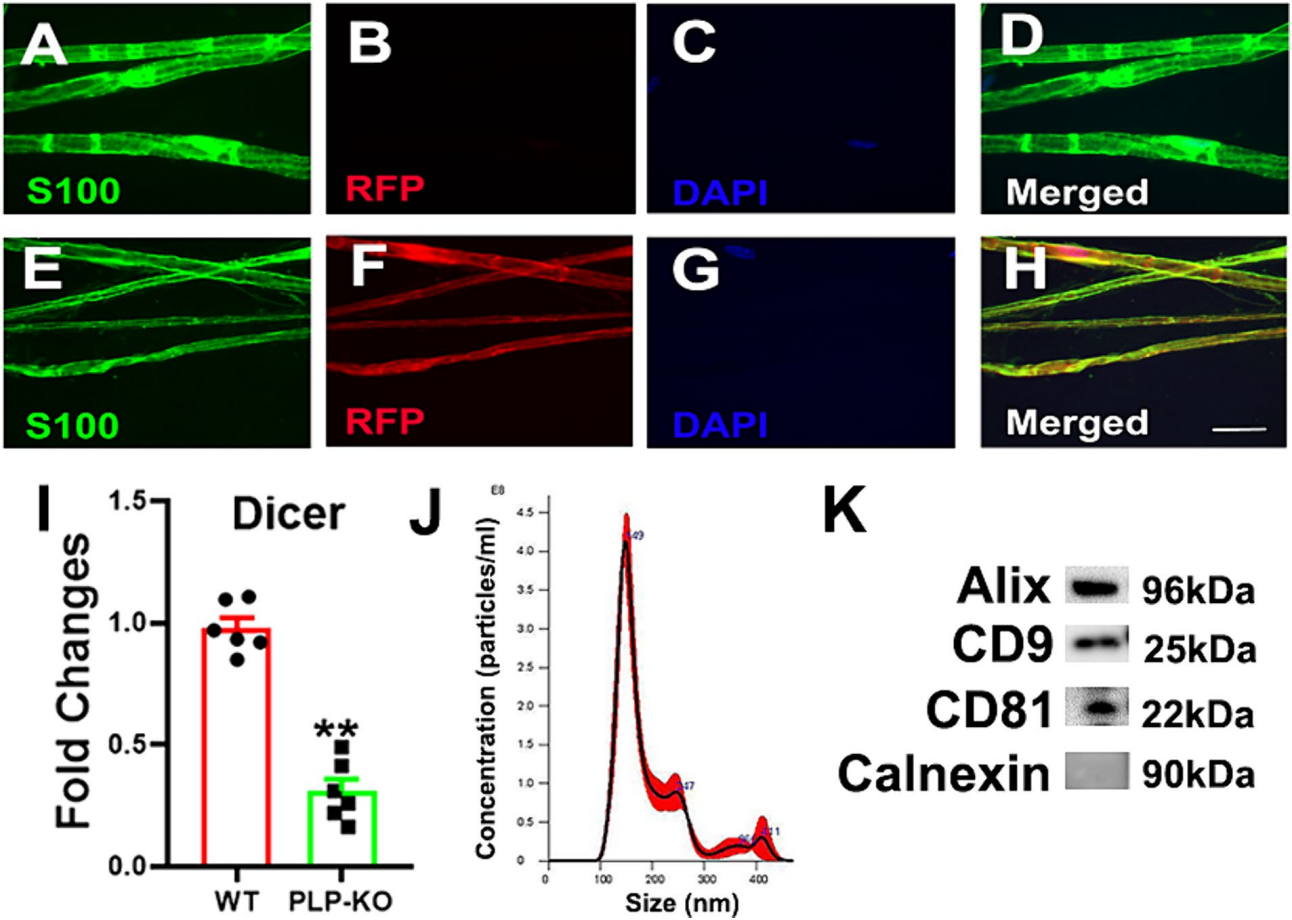
Characterizations of PLP expressing SC reporter and PLP-cKO mice and SC-Exo. **(A–H)** Show representative confocal images of teased sciatic nerve fiber from WT **(A–D)** and PLP-reporter mice **(E–H)**. In WT mice, there was no RFP signal, whereas RFP was co-localized with S100 immunoreactive cells in PLP-reporter mice. Scale bar in H = 20 µm. *n* = 3/group. **(I)** Shows that the levels of Dicer mRNA were significantly decreased in the sciatic nerve of PLP-cKO mice compared to WT mice. n = 6/group. Two-tailed Student’s t-test was used for two-group comparisons. **p < 0.01 versus WT mice. Error bars indicate the standard error of the mean. **(J**,**K)** Show size and distribution of SC-Exo measured by NanoSight **(J)** and exosomal marker proteins Alix, CD9 and CD81, and the negative exosomal marker protein Calnexin **(K)**. *n* = 3/group.

Using 8 week old PLP reporter female and male mice, we found that administration of TAM resulted in induction of red fluorescent protein (RFP) which was co-localized to S100 immunoreactive SCs in the sciatic nerves, while the treatment of age-matched WT mice with TAM did not induce RFP (Figure 1), indicating that PLP^-CreER^ induces specific and efficient recombination in SCs. We then analyzed Dicer mRNA in the sciatic nerve tissues of PLP-cKO mice 4 weeks after TAM treatment. Quantitative RT-PCR analysis revealed that compared to WT mice, Dicer mRNA was significantly reduced (Figure 1). Together, these data confirmed that Dicer is selectively knocked down in PLP-SCs.

**Figure 2:**
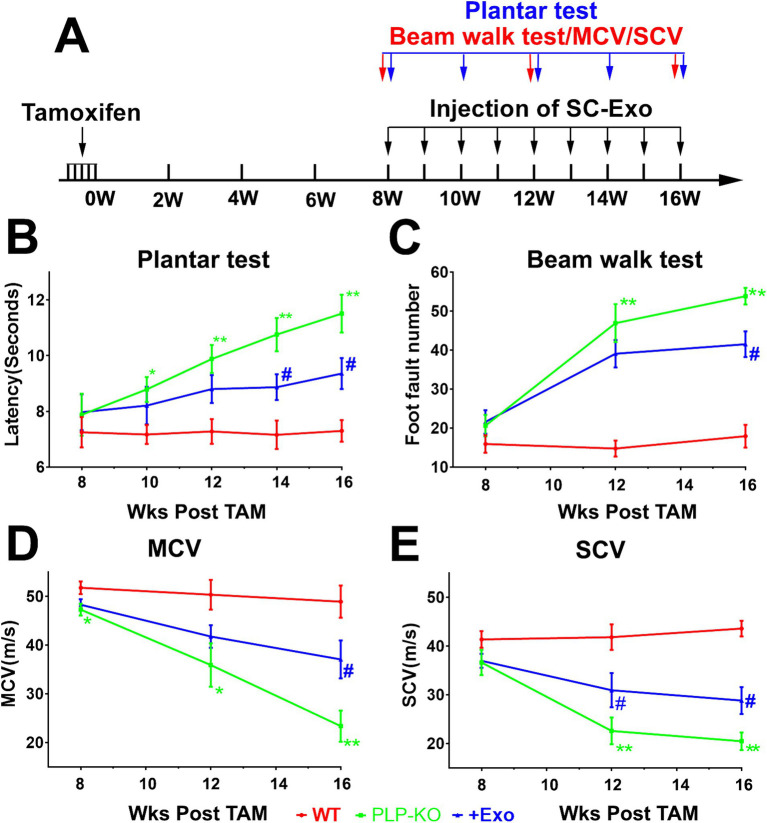
SC-Exo improve neurological function in PLP-cKO mice. A schematic **(A)** shows the experimental protocol. Treatment of PLP-cKO mice with SC-Exo (+Exo, blue, *n* = 12 mice/group, with 6/sex), improves neurological function measured by Plantar test **(B)**, Beam Walk test **(C)**, MCV **(D)** and SCV **(E)**, respectively. The repeated measure analysis of variance (ANCOVA) was used on each functional tests *p < 0.05, **p < 0.001 versus the WT mice treated with saline (WT, red, *n* = 12 mice/group, with 6/sex) and #*p* < 0.05, ##*p* < 0.001 versus the PLP-cKO mice treated with saline (PLP-KO, green, *n* = 11 mice/group, with 6 females and 5 males), respectively. Error bars indicate the standard error of the mean.

Next, we examined the peripheral neurological function of PLP-cKO mice. One PLP-cKO mouse died at 10 weeks after TAM, which was excluded from the present study. Eight weeks after TAM, PLP-cKO mice started to show impairments of motor coordination (Beam Walk test), thermal sensitivity (Plantar test), and sciatic nerve MCV and SCV, but the impairments did not reach statistical significance compared to WT mice, except for MCV (Figure 2). However, 12-week post TAM, PLP-cKO mice exhibited significant impairments in neurological function including loss of motor coordination, reduction of thermal sensitivity, and decreases of MCV and SCV compared to WT mice (Figure 2). Over the following 4 weeks (16 weeks after TAM), the impaired peripheral neurological function in PLP-cKO mice progressively worsened (Figure 2). The sex did not affect neurological impairment in PLP-cKO mice (Figure 2).

**Figure 3:**
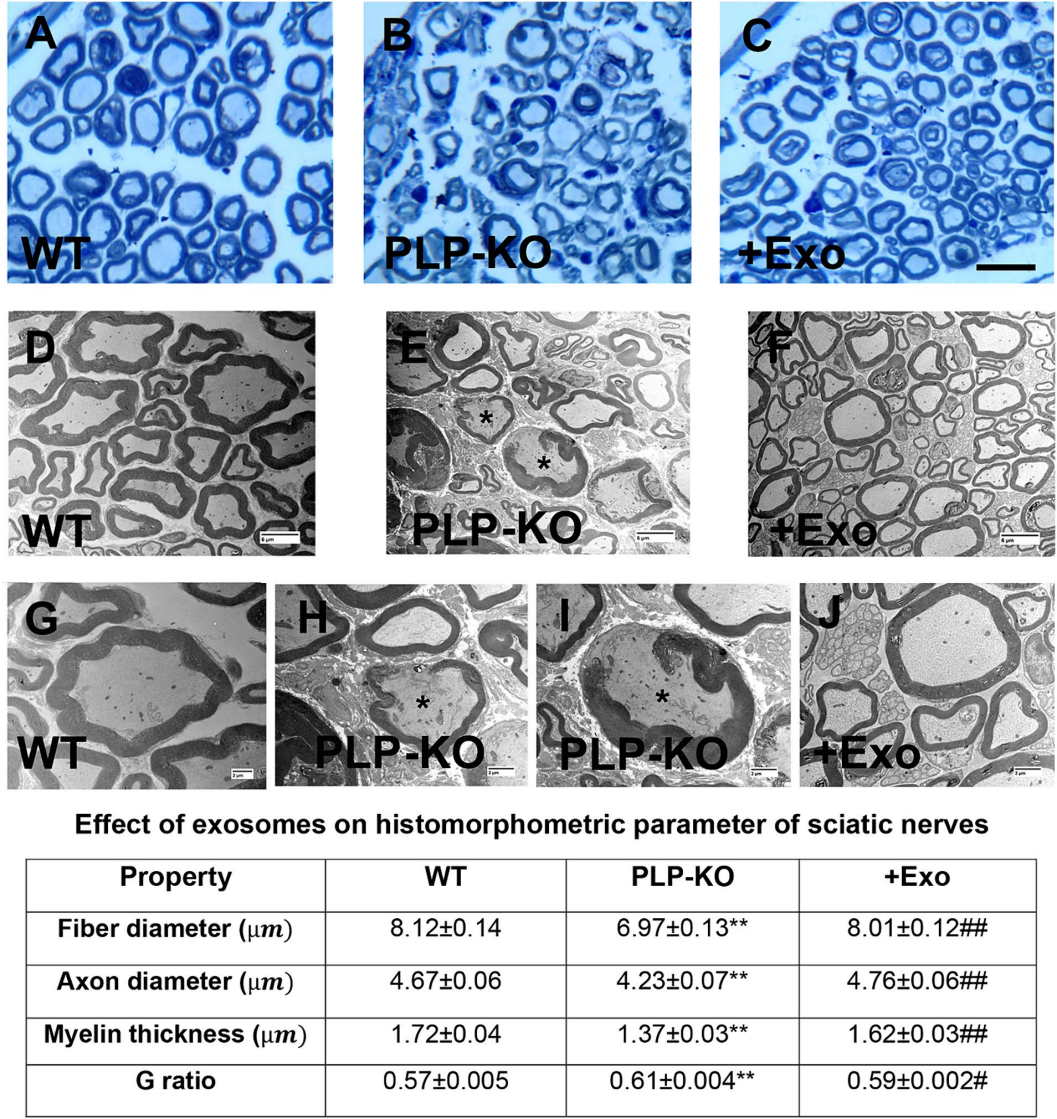
SC-Exo rescue the SC Dicer-ablation-induced histomorphometric changes of sciatic nerves. **(A**–**C)** Show representative images of semi-thin toluidine blue-stained cross sections of sciatic nerves from WT mice (**A**, WT), PLP-cKO mice treated with saline (**B**, PLP-KO), and PLP-cKO mice treated with SC-Exo (**C**, +Exo). **(D**–**J)** Show TEM image of ultrastructure of sciatic nerve from WT mice (**D**,**G**, WT) and PLP-cKO mice treated with saline (**E,H,I**, PLP-KO) and PLP-cKO treated with SC-Exo (**F**,**J**, +Exo). Demyelination, including discontinuous and disorganized myelin sheath, and damaged axons (asterisks in **E**,**H**,**I**) are observed in a PLP-cKO mouse. Table shows quantitative data on histomorphometric parameter of toluidine blue-stained of sciatic nerve. Scale bar: C = 25 μm, D-*F* = 2 μm, G-J = 6 μm. *n* = 8 mice/group. One-way ANOVA followed by Tukey’s multiple comparisons tests were used. **p < 0.01 versus WT treated with saline. #*p* < 0.05, ##*p* < 0.01 versus PLP-cKO treated with saline. Error bars indicate the standard error of the mean.

**Figure 4:**
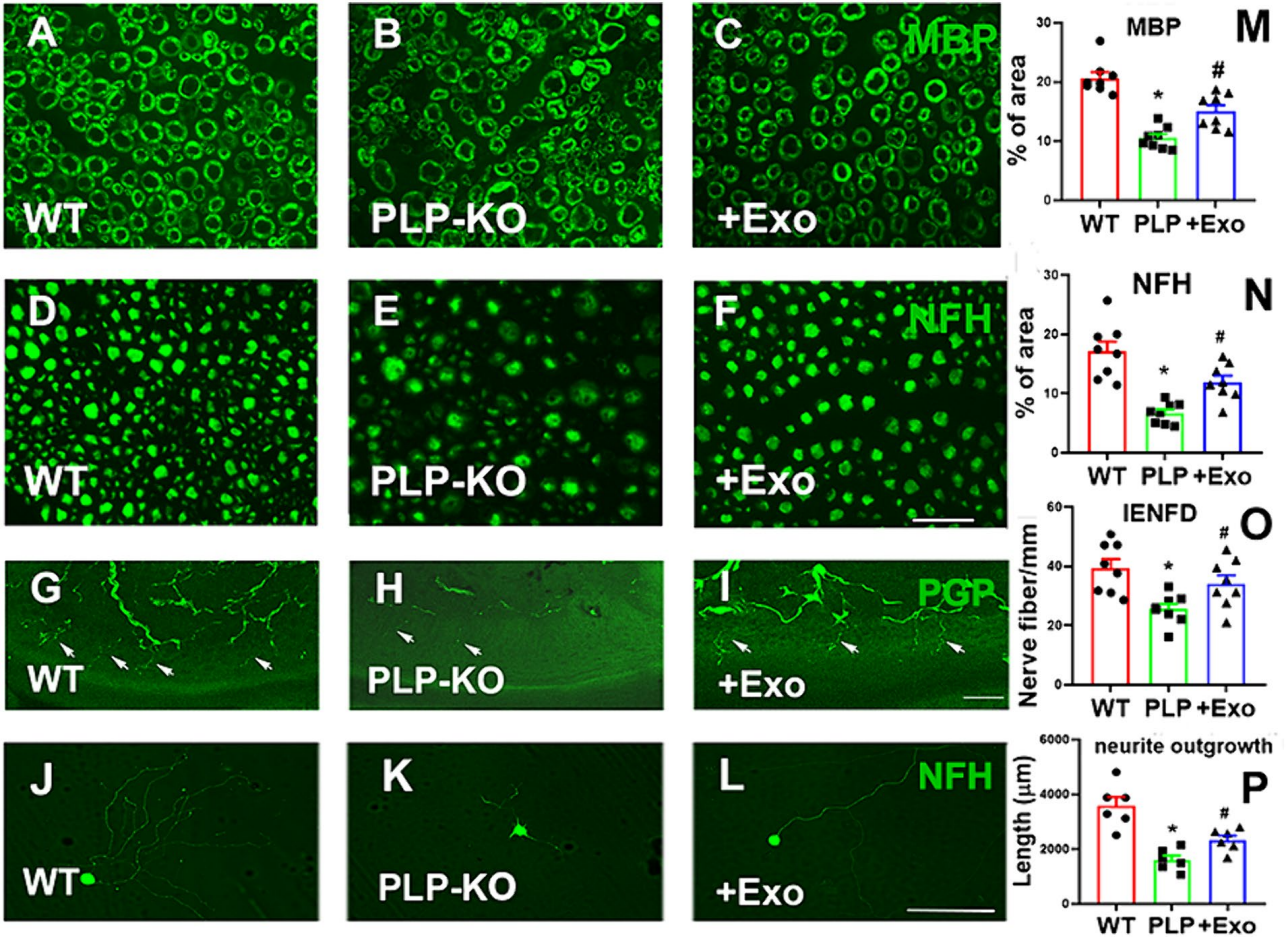
SC-Exo rescue the SC Dicer-ablation-induced morphometric changes of sciatic nerves in PLP-cKO mice. **(A–F)** Show representative images of MBP (**A-C**, green) and NF-H (**D–F**, green) immunoreactive sciatic nerves and PGP immunoreactive intraepidermal nerve fibers (IENF) in the hind plantar paw skin (**G–I**) from WT mice treated with saline (**A,D**,**G**, WT), PLP-cKO mouse treated with saline (**B,E**,**H**, PLP-KO), and PLP-cKO mouse treated with SC-Exo (**C,F,I**, +Exo) 8 weeks after treatment. The histogram represents the quantitative data of the percentage of MBP **(M)** and NF-H **(N)** immunoreactive area and IENF density **(O)**. *n* = 8 mice/group. Scale bar in *F* = 25 μm, *I* = 50 μm. **(J–L)** Show that representative microscopic images of neurofilament H (NFH) + neurite of DRG neurons derived from WT mice (WT, **J**), PLP-cKO mice (PLP-KO, **K**), PLP-cKO mice treated with SC-Exo (+Exo, **L**). **(P)** Shows quantitative data of neurite length of DRG neuron under different culture conditions. Scale bar in L = 50 μm. *n* = 6 mice/group. One-way ANOVA followed by Tukey’s multiple comparisons tests were used. *p < 0.05 versus WT treated with saline. #*p* < 0.05 versus PLP-cKO treated with saline. Error bars indicate the standard error of the mean.

Histopathological analysis of the sciatic nerve showed that compared to age-matched WT mice, PLP-cKO mice at age of 24 weeks (16 weeks post TAM) showed substantial and significant reductions in nerve fiber diameter and myelin sheath thickness, and significant increases in g-ratio (axon diameter/fiber diameter) (Figure 3). Ultrastructural analysis showed demyelination, including discontinuous and disorganized myelin sheath, and aberrant axonal morphology of the sciatic nerve in the PLP-cKO mouse (Figure 3). Additionally, PLP-cKO mice showed significant reductions of MBP positive myelin structure proteins and NF-H positive nerve fibers in the sciatic nerve, as well as PGP 9.5 positive intraepidermal nerve fibers (IENF) (Figure 4).

Moreover, cultured DRG neurons harvested from PLP-cKO mice 16 weeks after TAM exhibited a significant reduction of neurite outgrowth compared to DRG neurons from age matched WT mice (Figure 4).

Collectively, these data suggest that Dicer in adult PLP expressing SCs is essential to maintain peripheral nerve function, which is consistent with studies published by others (Viader et al., 2011; Li et al., 2018).

### SC-Exo improve neurological function in PLP-cKO mice

We examined whether administration of SC-Exo to PLP-cKO mice rescues the impaired peripheral neurological function. Prior to the treatment, SC-Exo were fully characterized (Thery et al., 2018; Wang et al., 2020) (Figure 1). Based on the functional data showing that PLP-cKO mice started to exhibit peripheral neuropathy at 8 weeks after TAM (Figure 2), the time point of 8 weeks post TAM was selected for the SC-Exo treatment. SC-Exo (2×10^10^ particles/mouse) were weekly administered (i.v) to PLP-cKO mice for 8 consecutive weeks. Compared to PLP-cKO mice treated with saline, PLP-cKO mice treated with SC-Exo exhibited significant improvements of motor coordination by 23% and thermal sensitivity by 19%, and increased the MCV by 59% and SCV by 41% at the end of 8 week SC-Exo treatment (Figure 2).

Treatment of PLP-cKO mice with SC-Exo significantly increased nerve fiber diameter and myelin sheath thickness, and reduced g-ratio compared to the saline treatment (Figure 3). Ultrastructural analysis of the sciatic nerve revealed that the SC-Exo treatment reduced demyelination and axonal damage, and augmented remyelination (Figure 3). Immune-histological analysis revealed that SC-Exo rescued levels of MBP+ myelin structural proteins, NF-H+ nerve fibers, and IENF in PLP—of Dicer in cKO mice (Figure 4).

To assess the direct effect of SC-Exo on ablation SCs on DRG neurons, SC-Exo were applied to cultured DRG neurons harvested from PLP-cKO mice, and neurite outgrowth was measured. SC-Exo significantly augmented neurite outgrowth of DRG neurons from PLP-cKO mice compared to PBS (Figure 4).

Together, these data indicate that the SC-Exo rescue the SC Dicer-ablation-induced impairment of peripheral neurological function.

### SC-Exo alter dicer-related miRNA and protein profiles in sciatic nerve tissues

**Figure 5:**
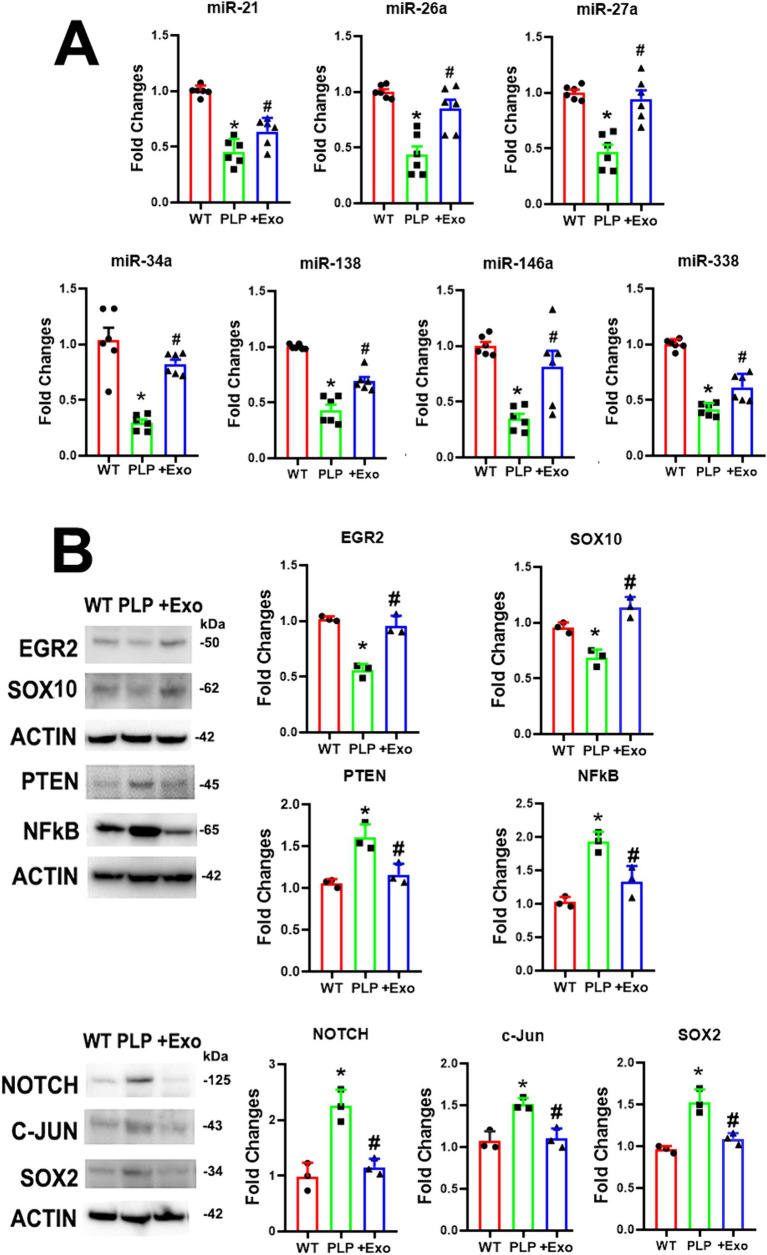
SC-Exo alter Dicer-related miRNA and protein profiles in sciatic nerve tissues. **(A)** Shows quantitative RT-PCR data of miR-21, −26, −27a, −34a, −138, −146a and − 338 levels in sciatic nerve tissue in WT mice treated with saline (WT), PLP-cKO mice treated with saline (PLP), and PLP-cKO mice treated with SC-Exo (+Exo). *n* = 6 mice/group. **(B)** Shows representative Western blot images and their quantitative data of protein levels of EGR2, Sox10, PTEN, NFkB, Notch, c-Jun and Sox2 in sciatic nerve tissue of WT mice treated with saline (WT), PLP-cKO mice treated with saline (PLP), and PLP-cKO mice treated with SC-Exo (+Exo). *n* = 3 mice/group. One-way ANOVA followed by Tukey’s multiple comparisons tests were used. *p < 0.05 versus WT treated with saline. #*p* < 0.05 versus PLP-cKO treated with saline. Error bars indicate the standard error of the mean.

miRNAs in SCs are crucial for maintaining myelination and the axonal integrity in the PNS (Pereira et al., 2010; Gokbuget et al., 2018). Based on published studies, we investigated the effects of SC-Exo on neuropathy related miRNAs: miR-21, −26a, −27a, −34a -146a, −138, and − 338 (Bremer et al., 2010; Viader et al., 2011; Wang et al., 2020). Using Taqman probes that specifically detect mature miRNAs, qRT-PCR analysis of the sciatic nerve tissue revealed that compared to WT mice, the ablation of Dicer in SCs resulted in a significant decrease in the expression of this set of miRNAs (Figure 5). The treatment of PLP-cKO mice with SC-Exo considerably elevated the levels of these miRNAs compared to the saline treatment (Figure 5). Interestingly, qRT-PCR analysis of SC-Exo cargo showed that SC-Exo were enriched with miR-21, −26a, −27a, − 34a, −138 and -146a, but not miR-338 (Table 1).

**Table 1:** miRNA expression in SC-Exo.

miRNAs	Average CT	SE (*n* = 3)
miR-21-5p	23.60	0.4
miR-27a-3p	28.24	0.3
MiR-146a-5p	28.99	0.2
miR-26a-5p	30.34	0.1
miR-34a-5p	31.77	0.3
miR-138-5p	34.50	0.1
miR-338-3p	39.25	0.9

SC-Exo cargo miRNAs were ranked based on CT values measured by quantitative RT-PCR and a high CT number represents a low level of miRNA. *n* = 3.

Western blot analysis revealed that compared to WT mice, myelin inhibitory proteins of c-Jun, Notch, and Sox2 were significantly increased, while myelin related proteins of EGR2 and Sox10 were considerably decreased in sciatic nerve tissues of PLP-cKO mice (Figure 5). Moreover, axonal inhibitory proteins of PTEN were significantly increased in PLP-cKO mice (Figure 5). The SC-Exo treatment significantly reversed these altered proteins (Figure 5). Ingenuity Pathways Analysis (IPA) showed that miR-21 and -26a can directly target Sox2 and PTEN, respectively, while Notch is potential target of miR-34a and miR-146a (Supplementary Figure S1). These data indicate that Dicer ablation in SC suppresses endogenous miRNA expression along with elevation of their putative target proteins, while SC-Exo reverse the altered miRNAs and proteins.

## Discussion

In this study, we demonstrated that intravenous administration of SC-Exo partially, but significantly rescued peripheral neurological function impaired by the conditional Dicer deletion in PLP expressing SCs in young adult mice, and that improved neurological function was associated with reduction of Dicer ablation-induced demyelination and axonal damage of the sciatic nerve. Moreover, treatment with exogenous SC-Exo reversed ablation of Dicer-altered miRNAs and proteins in the sciatic nerve tissues. These data suggest that SC-Exo have potential therapeutic effects on diseases related to Dicer impairment.

Although the effect of Dicer depletion in SCs on myelination and axonal damage has been studied, whether the exogenous exosomes impact the Dicer ablation-induced myelination and axonal impairments has not been investigated. Using *in vivo* and *ex-vivo* approaches, the present study for the first time demonstrated that the treatment of adult PLP-cKO mice with exosomes derived from healthy SCs, SC-Exo, ameliorated the neurological dysfunction along with reduction of demyelination and axon damage in peripheral nerves when the treatment started at the initial appearance of the neuropathy at 8 weeks post TAM. Our *ex vivo* data support our *in vivo* findings by showing that cultured DRG neurons harvested from PLP-cKO mice exhibited a significant reduction in neurite outgrowth, which was reversed by SC-Exo. Together, our findings suggest that SC-Exo reduce the severity and progression of Dicer ablation-induced peripheral neuropathy.

The ablation of Dicer in SCs leads to impairments of myelination, axonal integrity, and peripheral neurological function during development and in the adult animals (Pereira et al., 2010; Viader et al., 2011; Gokbuget et al., 2018; Li et al., 2018). However, the effect of Dicer ablation on myelination and axonal integrity varies. For example, Gokbuget et al. reported that ablation of Dicer in P0 lineage SCs results in impairments of maintenance of myelination with no impact on axonal integrity (Gokbuget et al., 2018), whereas Li et al. showed that inducible knockout of Dicer in adult PLP expressing SCs leads to massive sciatic nerve degeneration with peripheral motor dysfunction and paralysis, but without demyelination (Li et al., 2018). Interestingly, Viader et al. did not observe discernible pathology in peripheral nerves in PLP-cKO mice between 8 and 14 weeks post TAM (Viader et al., 2011). The present study demonstrated that conditional and inducible ablation of Dicer in PLP expressing SCs provokes peripheral neuropathy involving both motor and sensory nerves at 12-weeks post TAM and induces significant impairments of myelination and nerve fibers. In support the observed impairments, we found that depletion of Dicer in PLP expressing SCs resulted in robust reduction of the myelination essential protein, EGR2, and the myelin structural protein MBP, and a considerable augmentation of the axonal inhibitory protein, PTEN (Topilko et al., 1994; Wang C. et al., 2011; Ohtake et al., 2015). Moreover, ablation of Dicer in SCs led to increases in myelination inhibitory proteins, C-Jun, Notch, and SOX2 (Pereira et al., 2010; Yun et al., 2010). Importantly, we found that the SC-Exo treatment reversed these Dicer ablations altered proteins. Thus, the present study suggests that the Dicer ablation-altered proteins likely contribute to peripheral nerve dysfunction, which can be reversed by SC-Exo. Further investigations are warranted to determine how long the observed beneficial effects of SC-Exo in PLP-cKO mice persist when the 8 week treatment terminates, and whether delayed SC-Exo treatment of PLP-cKO mice will rescue Dicer ablation induced neuropathy.

The discrepancy between our findings and published studies on Dicer knock out mice are likely due to incomplete Dicer deletion, variations in specific experimental designs, and the analyses conducted. For example, in the present study, we noted that the severity of neurological and morphological impairments among PLP-cKO mice were varied, which is consistent with published studies reporting incomplete penetration of TAM induced Dicer ablation in inducible transgenic mice (Viader et al., 2011; Gokbuget et al., 2018).

Dicer is essential for the generation of mature miRNAs that play an essential role in the maintenance of adulthood myelination and peripheral nerve integrity (Pereira et al., 2010; Gokbuget et al., 2018). The ablation of Dicer in SCs results in reduction of Dicer-related miRNAs including miR-26a, −34a, −138, and −338 (Bremer et al., 2010; Viader et al., 2011). Consistent with published data (Bremer et al., 2010; Viader et al., 2011; Wang et al., 2020), the present study showed that deletion of Dicer in SCs significantly downregulated miR-21, −26a, −27a, −34a, −138, −146a, and −338 in the sciatic nerve tissues. These downregulated miRNAs likely contribute to the peripheral neuropathy observed in the PLP-cKO mice, because these miRNAs target myelin inhibitory proteins of c-Jun, Sox2 and Notch, and axonal inhibitory proteins of PTEN (Yun et al., 2010; Viader et al., 2011; Li and Sun, 2013; Sathyan et al., 2015).

The treatment of PLP-cKO mice with SC-Exo resulted in the recovery of Dicer depletion downregulated miR-21, −26, −27a, −34a, −138, −146a, and −338. Exosomes delivery their cargo miRNAs to recipient cells, leading to changes of recipient cell function (Bang and Thum, 2012; Kim et al., 2024). Our published study demonstrated that SC-Exo cargo enriched miR-21, −27a, and -146a suppress their target genes in recipient SCs of the sciatic nerve, resulting in improvements in DPN (Wang et al., 2020). The intravenously administered SC-Exo are internalized by the sciatic nerves (Wang et al., 2020). The present study showed that the treatment of PLP-cKO mice with SC-Exo resulted in the recovery of Dicer depletion downregulated miR-21, −26, −27a, −34a, −138, −146a, and −338, while many of the Dicer-downregulated miRNAs were enriched in the SC-Exo cargo. Thus, SC-Exo could transfer cargo miRNAs to the sciatic nerve, resulting in elevation of Dicer-ablation downregulated miRNAs. However, in addition to cargo miRNAs, other SC-Exo cargo materials such as proteins could also play a role to rescue dysfunctional neuropathy, as we have shown that SC-Exo contain many proteins that regulate myelination and axonal integrity (Wang et al., 2023). Additionally, SC-Exo could trigger SCs to upregulate miRNAs such as miR-338. Together, our data suggest that the therapeutic effect of SC-Exo on rescuing SC Dicer depletion induced peripheral neuropathy is a multifaceted process. It is warranted to additional investigations.

In the CNS, PLP is expressed in oligodendrocytes; thus, ablation of Dicer in PLP expressing cells likely impacts oligodendrocyte function. The effect of impairment of oligodendrocyte function on peripheral neuropathy and the therapeutic benefit of SC-Exo on oligodendrocytes in PLP-cKO mice warrant further investigation.

In summary, we demonstrated that exogenous SC-Exo reduces peripheral neuropathy induced by the ablation of Dicer in PLP expressing SCs, suggesting that SC-Exo are a potentially potent therapy to treat diseases with multiple dysfunctional miRNAs.

## Data Availability

The original contributions presented in the study are included in the article/Supplementary material, further inquiries can be directed to the corresponding author.
