# Supplementary material for: Schwann cell-derived exosomes ameliorate peripheral neuropathy induced by ablation of dicer in Schwann cells
Source: Front Cell Neurosci. 2024 Sep 2;18:1462228. doi: 10.3389/fncel.2024.1462228 (PMC11402728; doi:10.3389/fncel.2024.1462228)
Supplement: Supplementary file 1 [file Table_1.DOCX]

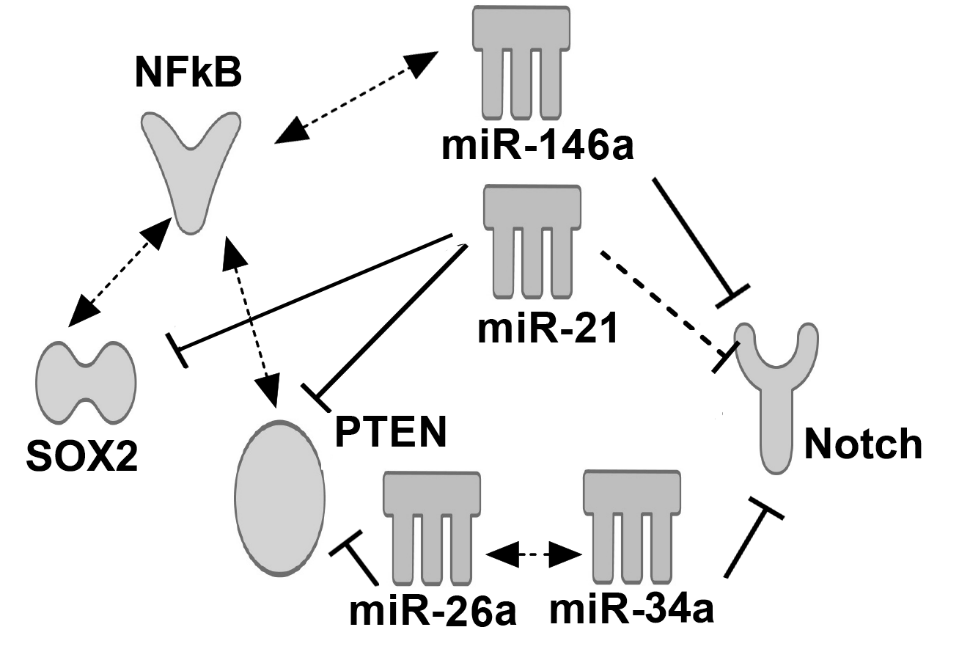


**Supplementary Figure 1.** The schematic shows the miRNAs/target genes networks that contribute to the impairment of myelination and axonal integrity in sciatic nerves. A solid line and a dashed line indicate direct and indirect targeting, respectively.
