# Supplementary material for: Schwann cell-derived exosomes ameliorate peripheral neuropathy induced by ablation of dicer in Schwann cells
Source: Front Cell Neurosci. 2024 Sep 2;18:1462228. doi: 10.3389/fncel.2024.1462228 (PMC11402728; doi:10.3389/fncel.2024.1462228)
Supplement: Supplementary file 2 [file Table_2.DOCX]

| **miRNAs** | **Company** | **Catalog number** |
| --- | --- | --- |
| hsa-miR-21-5p | ThermoFisher | 4427975 (ID#000397) |
| hsa-miR-26a-5p | ThermoFisher | 4427975 (ID#000405) |
| hsa-miR-27a-3p | ThermoFisher | 4427975 (ID#000408) |
| hsa-miR-34a-5p | ThermoFisher | 4427975 (ID#000426) |
| hsa-miR-138-5p | ThermoFisher | 4427975 (ID#002284) |
| hsa-miR-146a-5p | ThermoFisher | 4427975 (ID#000468) |
| hsa-miR-338-3p | ThermoFisher | 4427975 (ID#000548) |
| U6 snRNA | ThermoFisher | 4427975 (ID#001973) |

**Supplemental Table 1. miRNA primers used in real-time RT-PCR**
