# Supplementary material for: Schwann cell-derived exosomes ameliorate peripheral neuropathy induced by ablation of dicer in Schwann cells
Source: Front Cell Neurosci. 2024 Sep 2;18:1462228. doi: 10.3389/fncel.2024.1462228 (PMC11402728; doi:10.3389/fncel.2024.1462228)
Supplement: Supplementary file 3 [file Table_3.DOCX]

| Antibody name | Company and catalog number | concentration |
| --- | --- | --- |
| Anti-Alix | Cell Signaling, 2171 | 1:500 |
| Anti-CD9 | Abcam, ab92726 | 1:500 |
| Anti-CD81 | Abcam, ab109201 | 1:500 |
| Anti-Calnexin | Abcam, ab223052 | 1:500 |
| Anti-NOTCH | Cell Signaling,4380 | 1:500 |
| Anti-pNFkB | Cell Signaling,3033 | 1:500 |
| Anti-PTEN | Cell Signaling, 6559 | 1:1000 |
| Anti-CJUN | Cell Signaling,9165 | 1:500 |
| Anti-SOX2 | Cell Signaling,3728 | 1:500 |
| Anti-SOX10 | Cell Signaling,78330 | 1:500 |
| Anti-EGR2 | Santa Cruz, SC-20690 | 1:250 |
| Anti-β action | Abcam, ab6276 | 1:5000 |

**Supplemental Table 2. Antibodies used for Western Blots**
